# Progress in Machine Learning-Assisted Biosensors for Alzheimer’s Disease

**DOI:** 10.3390/bios16030161

**Published:** 2026-03-13

**Authors:** Yan Feng, Changdong Chen

**Affiliations:** 1 School of Artificial Intelligence, Pingdingshan University, Pingdingshan 467000, China; 6103@pdsu.edu.cn; 2 School of Chemistry and Environmental Engineering, Pingdingshan University, Pingdingshan 467000, China

**Keywords:** Alzheimer’s disease, electrochemical biosensors, optical biosensors, machine learning

## Abstract

Alzheimer’s disease (AD) is the most common cause of dementia, affecting 55 million people worldwide. Its characteristics include the accumulation of senile plaques and neurofibrillary tangles. This disease is associated with changes in the concentration of AD biomarkers, such as microRNAs, amyloid peptides, Tau protein, and neurofilament light chains. Due to the fact that neuropathological processes begin decades before the onset of cognitive symptoms, accurate detection of AD biomarkers is crucial for its early diagnosis. The combination of analytical techniques and machine learning methods plays a crucial role in medical innovation. Recently, efforts have been made to develop machine learning-assisted biosensors for AD diagnosis. This article provides an overview of the progress in machine learning-assisted sensing of AD biomarkers in bodily fluids. It mainly includes three parts: machine learning algorithms, machine learning-assisted electrochemical and optical biosensors, and challenges and future perspectives. We believe that this work will contribute to the development of innovative analytical devices based on artificial intelligence for monitoring and managing neurodegenerative diseases.

## 1. Introduction

Alzheimer’s disease (AD) is a neurodegenerative disease that accounts for 70~80% of dementia cases in the elderly. In 2017, over 120,000 AD patients died in the United States alone, and the cases increased by 145% between 2017 and 2000. This situation is worsening, with healthcare and hospice costs for patients aged 65 and above exceeding $290 billion each year [1]. The disease has affected 55 million people worldwide in 2025, and it is expected that the number will exceed 130 million by 2050 [2,3]. The main physiological symptom of this disease is the death of neurons in the AD brain. The neuronal death subsequently affects cognitive functions such as memory and behavior. In the later stages, AD has a significant impact on basic daily activities such as speaking and eating. Unfortunately, the identifiable symptoms of AD do not appear until 10 to 20 years after the onset of cellular degeneration. Therefore, strategies for early diagnosis of AD are crucial for successful medical interventions [4]. In order to develop intervention methods and prevent the spread of AD, researchers have been working hard to develop effective tools to diagnose the disease before the appearance of behavioral symptoms. So far, the diagnosis of AD is mainly based on the clinical manifestations, including patient history, cognitive tests, and medical examinations, followed by imaging and testing verification [5,6]. Nevertheless, these methods have the limitations of low early diagnosis rates, long diagnostic cycles, high costs, and strong invasiveness. It has been suggested that potential mechanisms for AD, such as amyloid-beta (Aβ) extracellular plaques and neurofibrillary tangles (NFTs), begin before the onset of clinical symptoms. The traditional methods for AD diagnosis mainly rely on clinical evaluation, exclusion diagnosis, and brain imaging. However, the accuracy of these diagnostic models is limited, especially in the mild cognitive impairment stage, making it difficult to distinguish between different types of neurodegenerative diseases. Moreover, through these methods, AD patients are usually diagnosed only when they show significant brain atrophy and cognitive dysfunction, which can easily cause them to miss the optimal intervention and treatment period.

Biosensors are analytical techniques that can distinguish and determine analytes with biological components. They have been widely developed to determine various compounds in fields such as food safety, environmental testing, and healthcare. The application of biosensors in point-of-care testing provides multiple advantages, such as less sample demand, on-site screening, and cost-effectiveness [7]. The combination of analytical techniques and machine learning methods plays a crucial role in medical innovation, which can be attributed to several important factors [8,9]. Firstly, machine learning-assisted biosensors can significantly improve the sensitivity, accuracy, and efficiency of point-of-care testing. These algorithms are capable of processing large amounts of complex biological data, enabling biosensors to accurately distinguish and interpret even small changes in analytes. Enhanced sensitivity is crucial for early detection of diseases, enabling healthcare professionals to identify issues at the optimal intervention time. In addition, improved accuracy can reduce the possibility of false positives or negatives and thus escape unnecessary medical interventions and missed diagnoses. This precision is particularly important when diagnostic test results have a significant impact on medical decision-making. Secondly, machine learning-assisted biosensors have fast data-processing capabilities and can provide almost real-time results, promoting rapid clinical decision-making and more effective treatment. Thirdly, machine learning technologies have multifunctionality and adaptability, which can provide solutions for various diagnostic problems. Machine learning models can significantly enhance the diagnostic capabilities of biosensors by classifying complex datasets, conducting regression analysis, and discovering patterns and trends in big datasets. Last but not least, integrating machine learning with biosensors for point-of-care testing can not only improve diagnostic accuracy and validity but also enable real-time detection, personalized therapy, and rapid response to medical emergencies.

Due to the lack of ideal biomarkers, the diagnosis of AD has mainly relied on invasive and traumatic methods, including autopsy or biopsy. For early diagnosis, medical history, laboratory testing, brain imaging, and psychological assessment have been considered. Artificial intelligence and machine learning have significantly promoted the development of a variety of methods for analyzing large and complex data, offering tools and advancing the field of AD diagnosis [10,11]. Many reviews discussed the advancement and prospects of artificial intelligence and machine learning in AD diagnosis; however, most of them exclusively focus on learning methods and architectures and/or specific data types. For example, several groups have discussed the potential application and diagnostic accuracy of different artificial intelligence models for AD [12,13,14,15,16,17], whereas others have addressed the techniques (e.g., positron emission tomography, magnetic resonance imaging, electroencephalography, and computed tomography) for diagnosing neurodegenerative diseases, including AD, with the assistance of machine learning algorithms [18,19,20,21,22,23,24]. Biomarkers, including nucleic acids and proteins, show great potential in the early diagnosis of AD [25,26,27]. Machine learning algorithms have been successfully used to assess some AD biomarkers available within cohort databases from hospital and community samples [28,29]. Although some excellent reviews have discussed the progress of sensing technologies for determining the concentration change in potential cerebrospinal fluid and blood AD biomarkers, including microRNAs, Aβ species, total and phosphorylated Tau proteins, and neurofilament light chains (NFLs) [30,31,32,33,34,35,36,37,38,39]. There is no specific review that systematically addresses the advancement of machine learning-assisted biosensors for AD diagnosis. This article aims to explore the development and applicability of machine learning-assisted biosensors in predicting the specification of different biomarkers for AD diagnosis. It emphasizes the collaborative combination of machine learning and biosensors, rather than addressing them as separate domains. The included studies until December 2025 were searched by Web of Science with the keywords of machine learning and electrochemical or different optical sensing techniques. The review is mainly divided into the following three sections: (1) general explanation of different machine learning algorithms, (2) systematic summary of the progress of various machine learning-assisted biosensors for AD diagnosis, and (3) brief addressing of the future development directions of machine learning-assisted biosensors.

## 2. Machine Learning Algorithms

**Scheme 1:**
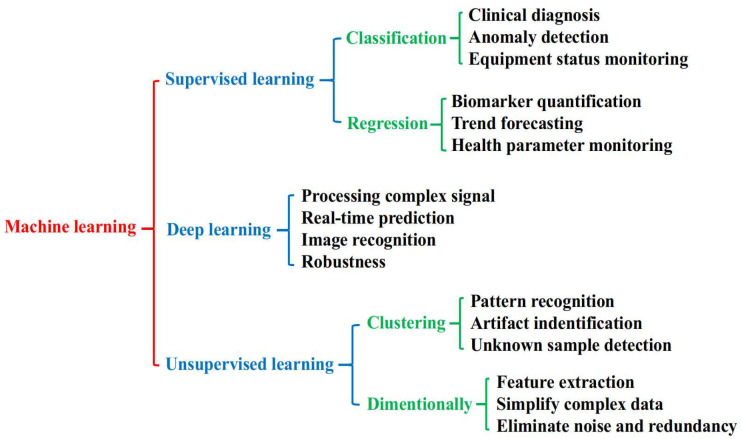
Different machine learning techniques and their functions.

Various machine learning algorithms have emerged with unique operational concepts and techniques [40,41]. Each machine learning algorithm method aims to solve the specific problem and tendency in rich data. In machine learning-assisted biosensors, choosing a perfect algorithm according to the signal characteristics and specific application is decisive for performance optimization. The optimum algorithm will improve various stages of data processing, including dimensionality reduction, feature extraction, anomaly detection, classification, and prediction [42]. Through different machine learning techniques, signals can be effectively classified or analyzed by regression, resulting in more reliable and informative diagnostic results in clinical environments. Scheme 1 shows an overview of machine learning methods in the application field of biosensing. Supervised, unsupervised, and deep learning techniques each provide different functionalities, ranging from diagnosis and anomaly detection to pattern recognition and real-time prediction [43]. As no algorithm is universally optimal, it is crucial to understand the principles and capabilities of various machine learning methods in order to select the most suitable solutions and obtain the best results for specific sensing applications [44]. This chapter briefly addresses several machine learning algorithms used for AD diagnosis by identifying biomarkers and outlining their functions to improve applicability in health monitoring, mainly focusing on supervised and deep learning methods.

### 2.1. Supervised Learning

Supervised learning methods can learn functions that map input to output based on labeled training data. A training dataset with input-output pairs can enable the methods to recognize the pattern and relationship. Usually, the datasets can be divided into training and testing subsets. General supervised learning tasks include classification and regression. The following section briefly introduces various supervised learning methods integrated with biosensors for AD diagnosis. Each algorithm has its own advantages and limitations. The choice of algorithm depends on the characteristics of the data and the specific problem that needs to be solved.

Linear regression is a simple machine learning algorithm that is designed based on multiple data points. The linear curve closest to these points can be obtained through regression. The curve can be used to predict results or understand the relationships between different factors. The advantages of linear regression lie in its simplicity, high computational efficiency, and strong interpretability. The model is suitable for the sample analysis with a simple matrix and minimal interference, such as standard solution calibration, pure substance quantitative assay, etc. It can replace traditional manual linear fitting curves with improved detection accuracy and efficiency.

A decision tree model combines various conditions widely used in regression analysis and classification. Random forest (RF) is based on multiple decision trees to employ ensemble learning methods for prediction. This model is generally applied to alleviate overfitting problems, where it works well on the training dataset but poorly on the testing dataset. The method can improve model performance through bagging and augmentation techniques. During the training process, multiple decision trees are required for random forest, and voting or averaging concepts are used for classification and regression predictions. In the classification model, the random forest predicts each decision tree for new points. The category with the highest frequency of occurrence is considered the final prediction result. In the regression model, each decision tree will predict a result for the new input. The final prediction value is the average of the predicted results of all individual decision trees.

AdaBoost, also known as adaptive reinforcement, constructs available and perfect models by integrating predictions from weak decision trees. The sequence ensemble model is usually applied in AdaBoost to transform weak learners into strong learners through weighting techniques. For each training data point, a specific weight is assigned. Then, these weights are used to train each hypothesis during the learning process. The purpose is to increase the importance of misclassified data by assigning higher weights, while reducing the importance of correctly classified data by assigning lower weights.

Gradient boosting is another ensemble learning algorithm that can be employed for classification and regression predictions. This method is based on the continuous building of several weak learners such as decision trees. Each decision tree can correct the mistakes in the previous trees and ultimately build a powerful predictive pattern. The additive AdaBoost model is required to identify the residuals of the previous model by assigning high weights to the data points with large errors. Gradient boosting can identify the mistakes in early models by gradients. The weights of all decision trees in gradient boosting are the same. However, the predictive efficiency will be limited by the learning rate in order to enhance the overall performance of the model.

The k-nearest neighbor (KNN) algorithm is a simple and powerful distance-based machine learning method used for classification and regression. In the case of classification, it assigns category labels to new data points based on the majority class among the KNN. In the case of regression, the KNN model makes predictions through the following steps. It first selects k neighbors and uses Manhattan distance or Euclidean distance to measure the distance between the testing data point and the training data point. By sorting the calculated distances in ascending order, the nearest data point can be determined. To predict the values corresponding to the testing points, the method ultimately calculates the mean of these neighbors.

A support vector machine (SVM) is a multifunctional machine learning model used for classification and regression. The SVM method involves the following five steps: (i) input data—receiving each training data point with a category label; (ii) feature space transformation—transforming input data points into a space with more dimensions and use kernel functions to complete this transformation; (iii) identifying hyperplanes—finding the most effective hyperplane in the transformation space and dividing the data into different categories; (iv) handling nonlinearity—mapping data to a larger dimensional space, enabling it to process non-linear relationships between different features; (v) prediction—in the case of classification, determining which side of the hyperplane a new data point will fall on. The side where the hyperplane is located determines the assigned category. In the case of regression, it will predict values based on the position of data points relative to the hyperplane.

### 2.2. Deep Learning

An artificial neural network (ANN) is a computational model like the structure and function of human brain tissue. Deep learning is an ANN-based machine learning concept. It outperforms shallow machine learning models and traditional data analysis methods. ANN is a subset of machine learning algorithms that can recognize patterns, predict results, and perform various tasks. A neural network node is the fundamental component of the network. Different layers (e.g., input layer, hidden layer, and output layer) are employed to organize the nodes. The neuron in one layer is fully connected to that in the next layer. This connection can easily transmit information. Each node is assigned a weight, determining the strength of the connection. During the model training process, these weights can be readily adjusted to empower network performance.

A convolutional neural network (CNN) is a type of neural network primarily used for analyzing visual images. It can automatically learn the spatial hierarchy of features. This will make it highly effective in pattern identification, target detection, and other visible tasks. The convolutional, pooling, and fully connected layers are three important components in CNN models. A convolutional layer can extract the feature representation from input data through an activation function. The pooling layer is usually located between the convolutional layers, which can reduce the resolution of the feature map to achieve shift invariance, connecting each feature map to the corresponding map from the previous layer. After several convolutional and pooling layers, a fully connected layer will perform advanced inference by connecting every neuron in the current layer to all neurons in the previous layer. This generates the final output of the CNN, which can represent the numerical values of the regression task or the probabilities of different categories in the classification task.

### 2.3. Other Algorithms

Besides the aforementioned data processing methods, other innovative and integrated algorithms such as logistic regression with peak-sensitive elastic-net regularization (PSE-LR), elastic network logistic regression (E-LR), principal component analysis post linear discriminant analysis (PCA-LDA), SRS-Net, RADAR, SSNet, *PyFasma*, and *RamanSPy* may also be suitable to be integrated with biosensors for AD diagnosis [45,46,47,48,49,50,51,52]. For example, very recently, Huang’s group developed an optimized algorithm, PSE-LR, for spectrum analysis and a label-free molecular atlas of Alzheimer’s brain [45]. This method classifies spectra with high-dimensional compatibility, subtle signal sensitivity, and excellent interpretability by generating peak-informed feature importance maps. The entire workflow mainly consists of three parts: simulating or experimentally measuring the spectra of different analytes, dividing the spectra into different categories by training and adjusting PSE-LR with peak-sensitive regularization, and extracting the spectral feature importance map with peak sensitivity to explain the classification feature and reveal the compositional difference between samples.

In addition, unsupervised learning is a data-driven machine learning method that can analyze unlabeled datasets without human intervention. It is typically used to identify patterns, trends, and structures in data, as well as for exploratory analysis. Unsupervised learning methods, such as hierarchical clustering analysis, *k*-means algorithm, and principal component analysis, have been effectively applied to discover meaningful patterns and simplify complex datasets, improving interpretability and practicality. In this review, we did not discuss unsupervised learning algorithms since they have not been integrated with biosensors for AD diagnosis. Researchers can read impressive review papers to find the advancement in unsupervised machine learning [53,54].

## 3. Machine Learning-Assisted Biosensors for AD

### 3.1. Electrochemical Biosensors

By integrating the results into a database, a machine learning algorithm can analyze the data, identify the pattern, and build a model to transform the raw signal into meaningful information. The combination of analytical techniques with machine learning has changed the directions of various point-of-care testing biosensors, including electrochemistry, optics, and microfluidics [55,56,57,58]. The cutting-edge technologies provide unparalleled sensitivity, specificity, and accuracy for AD diagnosis. With the help of machine learning, electrochemical biosensors can quickly process large and complex datasets and sensitively identify small patterns that may be indicative of diseases [59,60]. This combination enables point-of-care testing biosensors to offer accurate and timely data, facilitating diagnosis and treatment decision-making. Several studies have demonstrated that the combination of machine learning and electrochemical sensing technology is highly effective in AD diagnosis, including voltammetry, electrochemiluminescence (ECL), and field-effect transistor (FET) [61,62,63,64]. Here are several works demonstrating innovative applications of electrochemical biosensors integrated with machine learning (Table 1). The collected examples suggest how integration can enhance the accuracy, sensitivity, and reliability of biosensors, bridging the gap between raw data and actionable clinical diagnostics.

**Figure 1:**
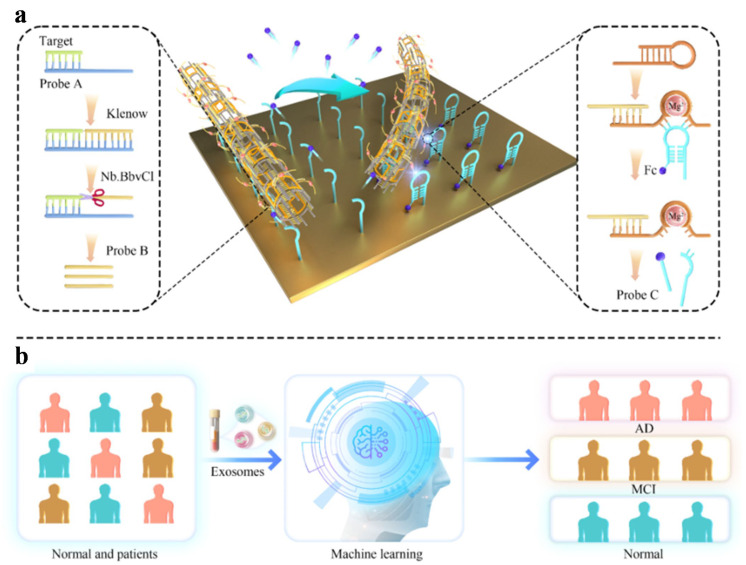
Schematic illustration of target-induced SDA to activate DNA nanotube for cleaving the immobilized probe (**a**) and machine learning analysis for AD diagnosis (**b**). Reprinted with permission from ref. [62]. Copyright 2025 Elsevier B.V.

Extracellular vesicle-encapsulated nucleic acids have emerged as prominent biomarkers for various diseases due to their earlier expression and resistance to nucleolytic degradation in biofluids. MicroRNAs encapsulated in exosomes become effective biomarkers for identifying mild cognitive impairment in neurodegenerative diseases. Cheng et al. proposed a high-speed machine learning-assisted electrochemical biosensor for decoding miR-193b from extracellular vesicles (Figure 1) [62]. In contrast to monovalent or divalent DNAzymes, the biosensor could accelerate reaction rates and attain high-gain signals by integrating a large number of DNAzymes into independent nanotube-shaped DNA structures. The DNA nanoharvester was successfully used to classify normal and APP/PS1 mice with a detection limit of 5.7 fM. With miR-193b as the diagnostic biomarker, the biosensor could identify normal individuals, mild cognitive impairment patients, and AD patients based on a linear discriminant analysis algorithm. In addition, Deshpande et al. reported an electrochemical diagnostic platform for AD diagnosis by monitoring the level of phosphorylated tau 181 (p-tau 181) in plasma and serum. Redox-active polyphenol red-based molecularly imprinted polymers were deposited on a porous gold electrode for the direct recognition of p-tau 181 with a detection of 980 fg/mL. A 100% classification accuracy with zero false positives/negatives was achieved based on the machine learning algorithm designed through a decision tree and RF. However, only 19 clinical samples were trained by the leave-one-out cross-validation method. For clinical applications, the vast majority of patients or non-AD donors are expected to avoid imbalanced distribution of categories in the datasets and provide prediction reliability and generalizability through the implementation of rigorous validation methods.

**Figure 2:**
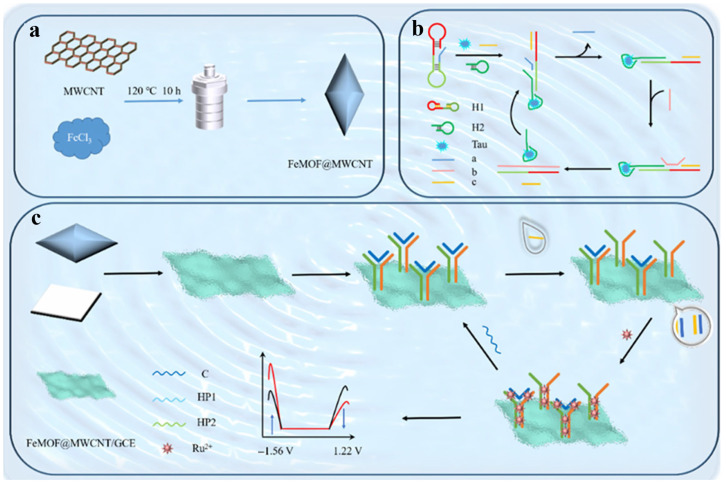
Schematic illustration of synthesis of FeMOF@MWCNTs (**a**), CHA process (**b**), and regenerative ratiometric ECL sensor for Tau protein detection (**c**). Reprinted with permission from ref. [63]. Copyright 2025 American Chemical Society.

In order to determine specific biomarkers, chemiluminescence (CL) and ECL sensing technologies have been used in point-of-testing devices based on the light emitted by chemical reactions [65]. In the CL system, samples are mixed with reagents such as luminol, hydrogen peroxide, and cobalt complex to produce light intensity by reacting with the targets. The working principle of the ECL system is similar, but it requires the use of current to trigger the chemical reactions. The ECL method can be employed to quickly and accurately collect the data, making it highly suitable for application in clinics or remote areas. Machine learning-assisted ECL system plays a key role in disease diagnosis by enhancing detection accuracy and effectiveness. They can interpret large and complex datasets, reduce reaction time, optimize measurement conditions, and improve signal collection capabilities, thereby enhancing the sensitivity and specificity. Yuan et al. reported a machine learning-assisted ratiometric ECL immunosensor for AD diagnosis by detecting Tau protein (Figure 2) [63]. In this work, Fe-based metal–organic framework (FeMOF) was used as the bidirectional regulator to modulate the dual-emission ECL signal from a single luminophore. The introduction of a bidirectional regulator into the ECL system effectively alleviated unstable fluctuations and small differences between the two signals, demonstrating stronger correlation and stability of signal ratios than that without regulation. The method could determine Tau protein with a detection limit of 3.38 fg/mL. Furthermore, it was used to identify normal and AD patients with 80% specificity and 90% sensitivity.

**Figure 3:**
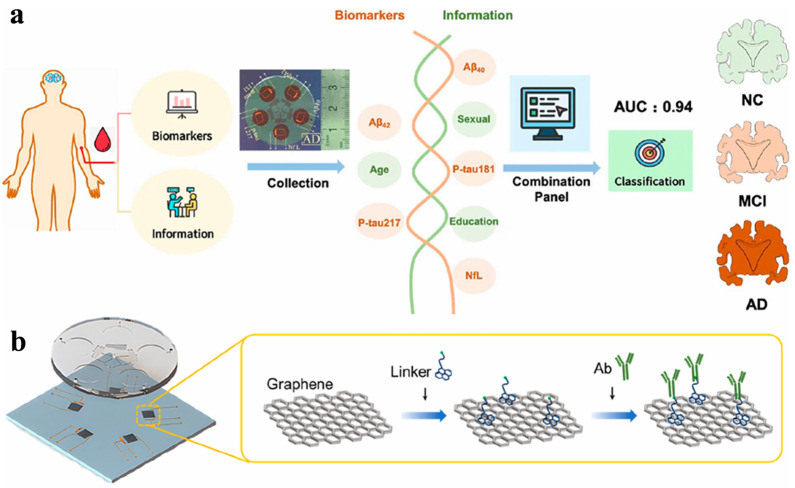
Schematic illustration of the biosensor for AD screening (**a**) and data acquisition process through gFET (**b**). Reprinted with permission from ref. [68]. Copyright 2023 American Chemical Society.

**Figure 4:**
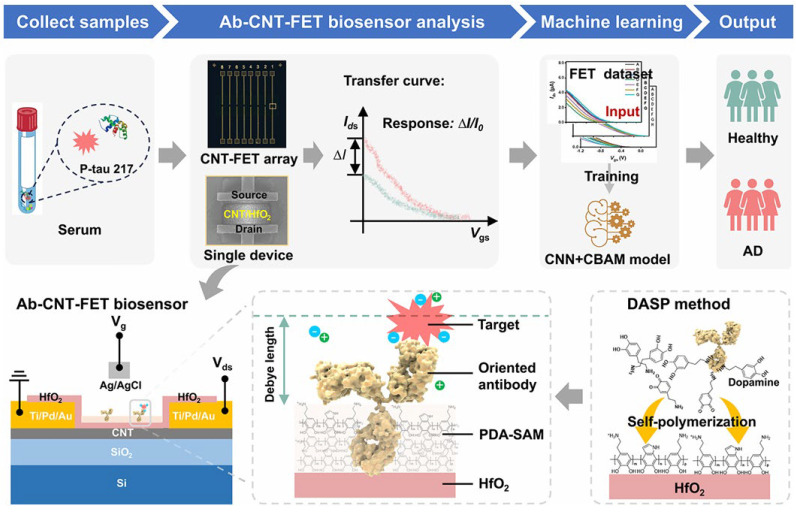
Schematic illustration of Ab-CNT-FET biosensor by DASP method for AD diagnosis. Reprinted with permission from ref. [69]. Copyright 2026 American Chemical Society.

FET is a voltage-controlled device type of transistor that can control current flow through a semiconductor channel using an electric field. FET-based biosensors have shown great potential in various fields such as healthcare, food, agriculture, environment, and military [66]. The biosensors can determine various biomolecules (e.g., proteins and nucleic acids) by utilizing the electrical properties of transistors. Multimodal integrations of FET with optical techniques have been proposed for the development of machine learning-assisted graphene and carbon nanotube-based platforms for the detection of multi-biomarkers [67]. Recently, Wang et al. developed a graphene FET biosensor for the detection of femtomolar AD biomarkers, including Aβ40, Aβ42, P-tau181, P-tau217, and NFL (Figure 3) [68]. The surface of a single graphene layer in a five-channel PDMS plate was modified with various target-specific recognition molecules. Machine-learning algorithms such as logistic regression, multilayer perceptron (MLP), and RF were used to analyze these biomarkers in human plasma, facilitating the discrimination of individual stages of AD progression. The AUC value was higher than 0.94 in the receiver operator characteristic curve. In addition, Gong et al. developed an FET biosensor for p-tau 217 detection with dopamine self-polymerization (DASP) strategy for antibody-oriented immobilization (Figure 4) [69]. The method involved the procedures of sample collection, FET analysis, machine learning, and diagnosis result output. The immobilization mechanism was based on the difference in the isoelectric point value between the two regions (Fc and Fab) of the antibody. Specifically, the relatively negatively charged Fc region facilitated the orientation immobilization through electrostatic and hydrophobic interactions with the amino group and benzene ring of dopamine. The positively charged region was confined away from the interface for target capture. The antibody-functionalized carbon nanotube-based FET (Ab-CNT-FET) biosensor could quantify p-tau 217 in the concentration range from fg/mL to ng/mL. By integrating the machine learning algorithm of CNN with the convolutional block attention module (CBAM), the diagnostic accuracy of this method reached 100% for the assays of 25 blood samples provided by 20 AD patients and 5 healthy donors.

**Table 1:** Overview of machine learning-assisted electrochemical biosensors for AD diagnosis.

Biosensor	Biomarker	Performance	Algorithm	Dataset/Validation	Ref.
SWV	miRNA-193b	5.7 fM LOD 87.5% precision 94.7% accuracy	LDA	19 clinical samples leave-one-out cross-validation	[62]
ECL	t-tau	3.38 fg/mL LOD 80% specificity 90% sensitivity	PLS-DA	20 serum samples	[63]
DPV	p-tau181	980 fg/mL LOD 100% accuracy	RF	undiluted plasma and serum	[64]
gFET	Aβ40, Aβ42, p-tau181, p-tau217, NFL	0.66 fg/mL LOD 85% accuracy	RF	66 clinical samples 5-fold cross-validation	[68]
FET	p-tau217	0.3 fg/mL LOD 100% accuracy	CNN	25 clinical samples 85% training and 15% test	[69]

Abbreviations: SWV, square wave voltammetry; LOD, limit of detection; LDA, linear discriminant analysis; t-tau, total Tau protein; ECL, electrochemiluminescence; PLS-DA, partial least-squares discriminant analysis; DPV, differential pulsed voltammogram; p-tau, phosphorylated Tau protein; gFET, graphene-based field-effect transistor; RF, random forest; FET, field-effect transistor; CNN, convolutional neural network.

The aforementioned methods highlight new opportunities for integrating machine learning with electrochemical techniques to enhance their accuracy and reliability in actual sample analysis. However, different analytes require unique sensing materials; selecting and integrating compatible materials to achieve stable and accurate detection remains a complex and intensive task. Realizing long-term material stability can ensure consistent sensor performance over time, which is crucial for maintaining the reliability of diagnostic and monitoring systems in clinical and nursing environments. Thus, the practical application of machine learning-based electrochemical biosensors is still in its infancy. Future research can focus on forecasting material stability and quality guarantee period by machine learning, increasing dataset size, and optimizing validation method, ensuring consistency and reliability of biosensors, and improving their generalizability in clinical applications.

### 3.2. Optical Biosensors

**Figure 5:**
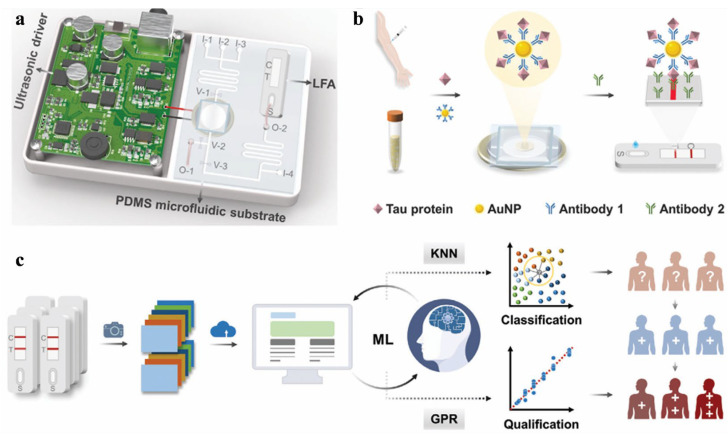
Schematic illustration of the integrated ultrasound-enriched LFA device (**a**), AuNP-based immunosensing strategy (**b**), machine learning-optimized LFA colorimetric analysis of tau protein and qualification for AD monitoring (**c**). Reprinted with permission from ref. [73]. Copyright 2024 Wiley-VCH.

Developing flexible optical biosensors has received widespread attention owing to their non-invasive nature, ease of observation, and ability for successive health monitoring. The biosensors significantly improve the accuracy and speed of disease monitoring, especially when combined with artificial intelligence [70,71]. This combination opens up new opportunities in continuous health monitoring and personalized medicine. Ultrasensitive detection of biological entities can be achieved through machine learning-assisted colorimetric, fluorescent, Raman scattering, and other optical biosensors (Table 2). In colorimetric assays, the signal transduction mechanism will display a color change when the target analyte binds to the sensor material, which is usually induced by redox reaction, enzyme catalysis, or surface change in the sensing materials. With the assistance of machine learning, colorimetric biosensors are becoming increasingly important in analysis fields and have been used for effective determination of various substances with minimal equipment investment and professional knowledge [72]. Wang et al. proposed a machine learning-assisted gold nanoparticles (AuNPs)-based colorimetric LFA for point-of-care diagnosis of AD by monitoring the level of Tau protein (Figure 5) [73]. The LFA device was combined with a portable ultrasonic actuator for enriching AuNPs by ultrasound, which is essential for sample pre-enrichment to improve sensitivity. By integrating with two machine learning algorithms (KNN and Gaussian process regression), the LFA platform could efficiently classify and quantify Tau protein with a detection limit of 10.30 pg/mL. The classification and prediction accuracies were found to be 98.11% and 99.99% for point-of-care testing of human plasma samples, respectively.

**Figure 6:**
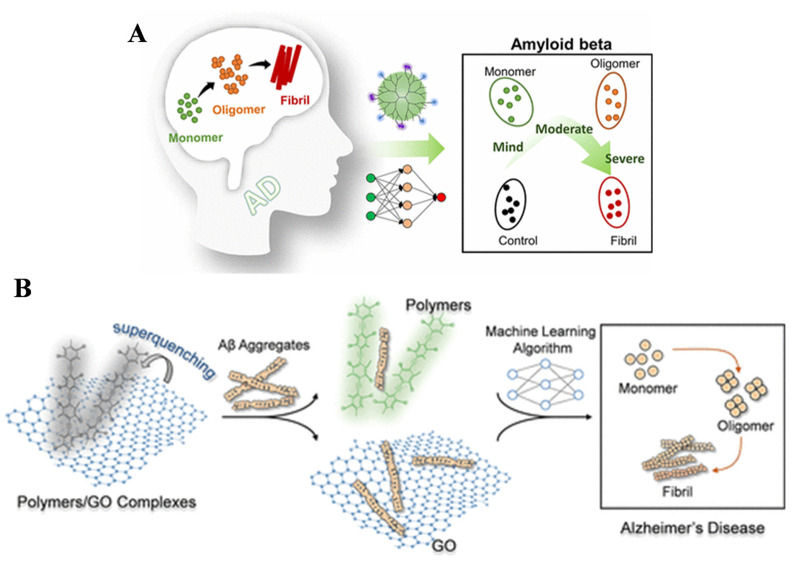
(**A**) Schematic illustration of the sensor array for identifying Aβ40/Aβ42 aggregates. Reprinted with permission from ref. [76]. Copyright 2022 American Chemical Society. (**B**) Schematic illustration of the electrostatic complexes and identification mechanism for multiple analytes. Reprinted with permission from ref. [77]. Copyright 2022 American Chemical Society.

Fluorescent probes have been widely used for the identification and diagnosis of disease-related events because of their high sensitivity, good selectivity, and excellent capability to integrate with imaging technologies. However, the probes currently used for early clinical diagnosis of AD are invasive, expensive, susceptible to interference from biological background fluorescence, and lack sufficient penetration capability and challenges related to in vivo imaging [74,75]. The analytical performance of fluorescent probes or biosensors can be improved by integrating with artificial intelligence to process data. Machine learning can filter out noise from raw signals, extract relevant features, and fully decode complex fluorescence parameters. The ensemble methods can classify signals into diagnostic categories such as positive, negative, or uncertain, and accurately distinguish the quantity of analytes. Xu et al. reported a fluorescent sensing array for the detection of multiple Aβ aggregates through machine learning algorithms (Figure 6A) [76]. The array includes three pyrene-modified polyamidoamine dendrimers. The modified functional groups allowed for the identification of 11 proteins with 100% accuracy through linear discriminant analysis. The biosensor showed good anti-interference ability and high accuracy for the assays of cerebrospinal fluid and serum samples. Three machine learning algorithms, including DT, SVM, and logistic regression, were used to distinguish different formats of Aβ40 and Aβ42 species (e.g., monomer, oligomer, and fibril). At the same time, Wang et al. reported five sensing probes for early diagnosis of AD based on machine learning algorithms (Figure 6B) [77]. The probes were prepared through the electrostatic interactions of fluorescent poly (*para*-aryleneethynylene) polymers and graphene oxide quencher. The method could be used to distinguish between 12 proteins with 100% accuracy. Machine learning algorithms including DT, RF, SVM, and LR were considered for the assays of Aβ40 and Aβ42 examples. As a result, the SVM and LDA methods obtained 100% accuracy.

**Figure 7:**
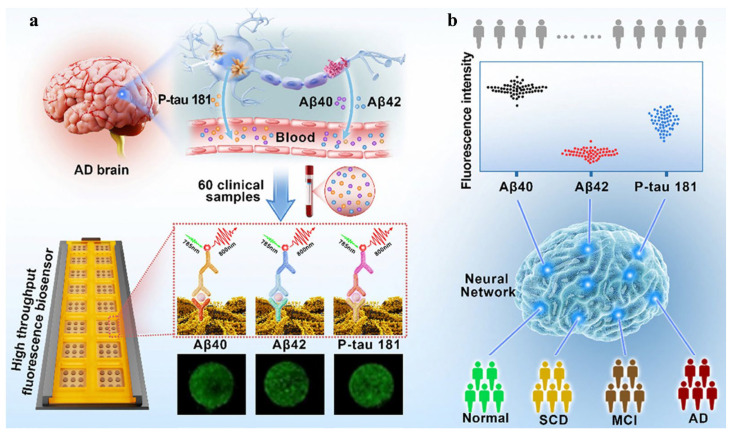
Schematic illustration of machine learning-assisted AD diagnosis grading system for fluorescent detection of three blood biomarkers. (**a**) Brain-derived biomarkers traverse into the bloodstream, and a multiplex fluorescent biosensor quantitatively analyzes these markers in blood samples. (**b**) ANN algorithms are employed to process the fluorescence signals from the three markers measured for AD diagnosis and grading. Reprinted with permission from ref. [78]. Copyright 2025 Royal Society of Chemistry.

Although blood biomarkers are becoming increasingly important for the diagnosis of AD, the diagnostic accuracy with a single biomarker still remains poor owing to individual differences. For this view, Liu et al. proposed a multiplex fluorescent immunosensing platform for the simultaneous detection of three AD biomarkers: Aβ40, Aβ42, and phosphorylated tau 181 (P-tau181) (Figure 7) [78]. The detection chip contains sixteen compartments. The compartment has nine spots arranged in three rows. ANN algorithm for the assays of 60 clinical blood samples achieved 91% predictive accuracy, 8.8% false rate, and >90% positive predictive value.

Raman spectroscopy can use inelastic light scattering to identify molecular vibrations and highly differentiate them based on the chemical properties of biological structures such as blood, saliva, urine, and tear liquid biopsies [79,80]. This technology is non-destructive and does not involve any chemical labeling. Thus, Raman spectroscopy can be directly applied to complex biological systems. With the help of artificial intelligence, raw Raman spectroscopy data can be input into machines, noise can be extracted, and patterns corresponding to biomarkers or disease states can be easily found. The machine learning algorithms can classify and interpret spectral information, facilitating the diagnosis in cases of complex or significant spectral overlap in the spectral regions. Raman scattering, especially surface-enhanced Raman scattering (SERS), has become an innovative optical biosensing technique. However, Raman signals are usually weak and susceptible to interference from noise and overlapping spectral features, making them difficult to analyze with traditional techniques. Machine learning algorithms are highly effective in processing high-dimensional datasets, essentially improving the capability of SERS biosensors. Here are several examples indicating the applications of Raman biosensors by combining with machine learning [81,82,83,84,85,86,87,88,89].

**Figure 8:**
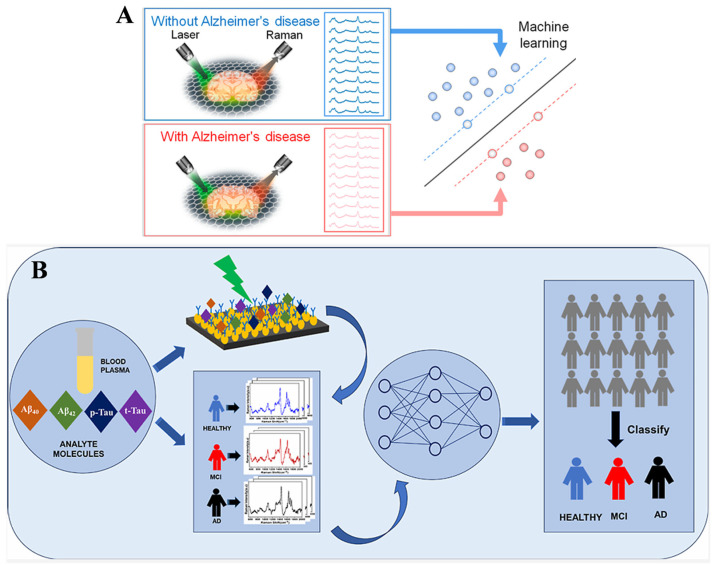
(**A**) Schematic illustration of workflow of graphene-assisted collection of Raman data, preprocessing, and machine learning classification and interpretation. Reprinted with permission from ref. [84]. Copyright 2022 American Chemical Society. (**B**) Schematic illustration of the SERS-based immunoassay platform for the detection of AD biomarkers. Reprinted with permission from ref. [85]. Copyright 2024 American Chemical Society.

Due to feature noise, model complexity, and lack of spectral optimization, traditional machine learning methods face challenges in interpretability for generating clear feature importance maps to highlight the spectral features unique to each class of data, especially in brain tissue [90,91]. Huang’s group reported a machine learning-assisted graphene-based Raman platform for identifying mouse brains with and without AD (Figure 8A) [84]. The brain slices were placed in monolayer graphene for the collection of Raman data. In this work, AD biomarkers, including Aβ and tau proteins and others (e.g., triolein, phosphatidylcholine, and actin), have been identified by the linear SVM method. The accuracy reached 98% through the machine learning classification. This integration facilitated the early diagnosis of AD and the identification of other tissues and biofluids from different diseases. The algorithm is comparable to other representative machine learning methods, including E-LR, SVM, PCA-LDA, tree-based model (XGBoost), KNN, and ANN. To improve the diagnosis accuracy and specificity, Resmi et al. developed an immuosensing SERS platform for the simultaneous detection of multiple core AD biomarkers (Aβ40, Aβ42, p-tau, and t-tau) through machine-learning algorithms (Figure 8B). The target-specific antibodies were immobilized on an aluminum SERS substrate. The SERS platform exhibited a wide dynamic range for target concentration, changing from attomolar to micromolar. It showed great potential in identifying blood plasma samples from mild cognitive impairment, AD, and healthy groups by integrating with MLP, RBF, SVM, and LDA algorithms.

**Figure 9:**
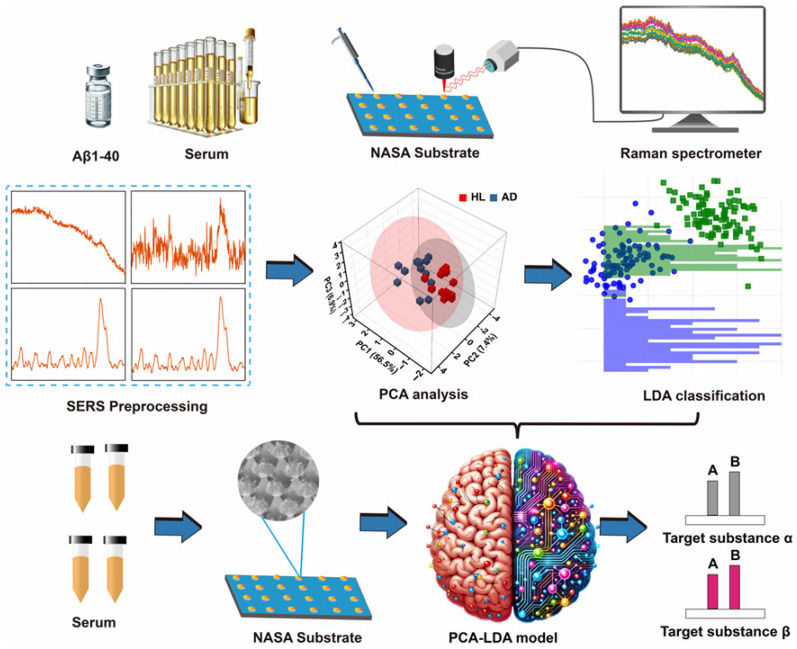
Schematic illustration of serum analysis platform combining machine learning and SERS techniques for identifying AD rats. Reprinted with permission from ref. [86]. Copyright 2024 American Chemical Society.

**Figure 10:**
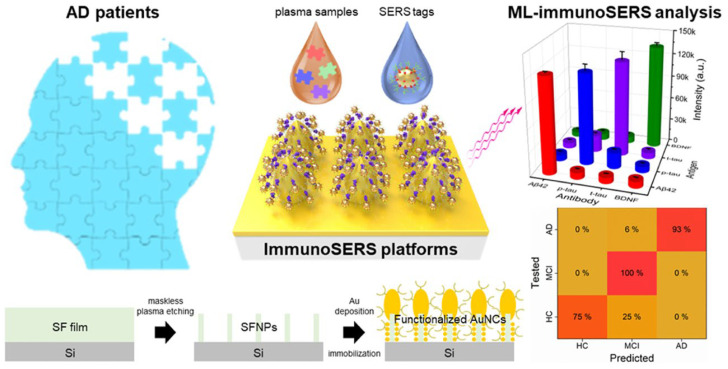
Schematic illustration of early AD diagnosis based on the SERS immunosensing platform. Reprinted with permission from ref. [89]. Copyright 2026 Elsevier B.V.

To further explore the application of machine learning in SERS spectra for AD identification, Lu et al. used a closely ordered Nano Stellar Array (NASA) as the SERS substrate to achieve the detection of Aβ40 in AD rats (Figure 9). A machine learning model was constructed through PCA–LDA algorithm. This method showed good performance in distinguishing the SERS spectra of AD serums. The accuracy, sensitivity, specificity, and area under curve (AUC) were found to be 95.8%, 100%, 92%, and 0.97, respectively. In addition, Kim et al. reported a deep learning-assisted SERS platform for the recognition of Aβ42 and metabolites. An Au nanowire array as the SERS substrate was prepared and functionalized in two different characteristic ways. In the first way, an anti-Aβ42 antibody was immobilized on the substrate surface for the assays of Aβ42 and its oligomerization process. In another way, different self-assembled monolayers were prepared to identify various dipole interactions with the metabolites in blood plasma. The accuracy reached to 99.5% for the assays of metabolites by advanced deep learning. The result suggested that an artificial intelligence-based SERS platform can be used to identify key spectral features for discovering and classifying potential biomarkers. Silk fibroin has been used as a natural etching mask. In order to enhance plasmonic hotspot formation for ultrasensitive analysis, Lee et al. designed a silk fibroin-templated SERS immunosensing platform for AD identification (Figure 10). With silk fibroin as the material to fabricate Au nanocavity (AuNC) SERS substrate, the method achieved attomolar detection limit and high reproducibility for the assays of four core AD biomarkers in human plasma (Aβ42, t-tau, p-tau, and brain-derived neurotrophic factor BDNF). Using a KNN-based machine learning algorithm, the biosensor could monitor the AD progression stage with 94% accuracy.

Surface plasmon resonance (SPR) biosensors integrated with artificial intelligence have the ability to accurately collect diagnostic signals [92]. In this method, a thin metal surface (usually gold) is irradiated using polarized light to excite a surface plasmon. The capture of biomolecules on the surface of functionalized chips can lead to a change in the refractive index. This can be monitored by the shift in the resonance angle. Machine learning algorithms can be used to filter data, determine the SPR signal and noise quality, and isolate relevant data [93,94]. The technique can distinguish small changes and display high sensitivity. Through the integration with artificial intelligence, the accuracy and efficiency of SPR biosensors can be significantly improved for the monitoring of multiple AD biomarkers. However, in complex biological environments, the actual deployment of a single-model biosensor is often hindered by signal fluctuation, matrix interference, and limited quantitative robustness [95,96]. To address these intrinsic limitations, multimodal sensing strategies have gained increasing attention by integrating SPR with complementary transduction modalities, enabling enhanced analytical reliability, internal cross-validation, and multidimensional biochemical profiling, including the combination with electrochemistry, colorimetry, fluorescence, SERS, and so forth.

**Table 2:** Overview of machine learning-assisted optical biosensors for AD diagnosis.

Biosensor	Biomarker	Performance	Algorithm	Dataset/Validation	Ref.
LFA	t-tau	10.3 pg/mL LOD 99.99% accuracy	KNN, GPR	6 clinical samples 5-fold cross-validation	[73]
Fluorescence	Aβ40/Aβ42 aggregates	0.5 μM LOD 100% accuracy	LDA	44 unknown samples leave-one-out cross-validation	[76]
Fluorescence	Aβ40/Aβ42 aggregates	5 μM LOD 100% accuracy	LDA	20 unknown samples leave-one-out cross-validation	[77]
Fluorescence	Aβ40, Aβ42, p-tau181	0.43 pg/mL, 0.68 pg/mL, 0.71 pg/mL LOD 91% accuracy	ANN	60 clinical samples 85% training and 15% test	[78]
SERS	Aβ oligomers	85% accuracy	t-SNE	20 AD and 11 ONC CSF samples 5-fold cross-validation	[83]
SERS	Aβ and Tau proteins	98% accuracy	SVM	mice brain slices 5-fold cross-validation	[84]
SERS	Aβ40, Aβ42, p-tau, t-tau	13.64 aM (Aβ42) and 28.6 aM (p-tau) LOD high accuracy	MLP, RBF, SVM, and LDA	60 clinical samples 70% training and 30% test	[85]
SERS	Aβ40	95.8% accuracy	PCA-LDA	AD and healthy rats 5-fold cross-validation	[86]
SERS	R6G	41.87 fM LOD 95% accuracy	PCA-WRKNN	mice serums leave-one-out cross-validation	[87]
SERS	Aβ42	87.5% accuracy	ANN	20 clinical samples 5-fold cross-validation	[88]
SERS	Aβ42, t-tau, p-tau, BDNF	35.8 aM LOD 94% accuracy	KNN	66 clinical samples 5-fold cross-validation	[89]
SERS	Aβ42, t-tau, and p-tau	92% accuracy	CNN	15 unknown CSF samples leave-one-out cross-validation	[97]

Abbreviations: LFA, colorimetric lateral flow assay; t-tau, total Tau protein; LOD, limit of detection; KNN, k-nearest neighbor; GPR, gaussian process regression; LDA, linear discriminant analysis; p-tau, phosphorylated Tau protein; ANN, artificial neural network; t-SNE, t-distributed stochastic neighbor embedding; ONC, other neurological conditions; CSF, cerebrospinal fluid; SVM, support vector machine; MLP, multilayer perceptron; RBF, radial basis function; PCA, principal component analysis; WRKNN, weighted representation-based k-nearest neighbor; BDNF, brain-derived neurotrophic factor; CNN, convolutional neural network.

Although machine learning-assisted biosensors have made progress in AD diagnosis, scientists and clinicians may still remain cautious due to the “black box” nature of learning models. This is also one of the main obstacles to the adoption of machine learning in the medical field. The lack of interpretability in predictive models may undermine trust in reporting models. No model is perfect, so people are tired of blindly believing that the predictions of a model that cannot provide any potential reasons for decision-making are completely reasonable. A common application of machine learning in AD diagnosis is for early warning systems to predict clinical deterioration. However, if such systems only warn clinical doctors of the risk of worsening the condition, the reason for issuing the alert may not be clear without further evaluation, which could delay treatment in time-sensitive situations or waste valuable clinical time in the case of false positives. In order to address the significant challenges posed by the “black box” model, there is an urgent need for interpretable research work. The main focus of this study is not to make “black box” models inherently interpretable, but to provide understandable explanations for how the model works and why specific personal predictions are made. The prospect of explainable machine learning is very promising: there is an opportunity to benefit from the state-of-the-art predictive capabilities of technologies such as deep learning without the drawbacks of “black box”. However, this commitment also has some important limitations. Key concepts and techniques applied in the field of interpretable machine learning can be found in some review articles [98,99].

## 4. Challenges and Future Perspectives

Compared with traditional diagnosis methods used in the laboratory, the reliability and accuracy of point-of-care testing biosensors are usually poor. The introduction of machine learning into biosensors provides a promising approach to improve the accuracy and reliability of point-of-care biosensors in the analysis of AD samples. The reported studies suggest that the combination of machine learning with electrochemical and optical biosensors can significantly improve the accuracy and specificity for AD diagnosis. The synergistic effect will not only accelerate the diagnostic process but also ensure reliable real-time data. This is very crucial for timely medical intervention. However, most of the biosensors involved in this study were developed for the determination of a single analyte. This will limit their practical application in early diagnosis of AD, as it is difficult to distinguish AD from other types of neurodegenerative diseases through a single biomarker. For this viewpoint, it is crucial to integrate multiple sensing methods, although there is a lack of standardized data formats and communication protocols between various biosensors. The diagnosis accuracy and reliability may be improved through the combination of multimodal biosensors with a unified detection system for the simultaneous determination of multiple AD biomarkers. In addition, in the early stages of research, machine learning-assisted diagnosis methods require a large amount of samples and data. Key issues such as data privacy, ethical considerations, and algorithmic biases require the implementation of robust encryption protocols, transparent data governance frameworks, and interpretable artificial intelligence models. Future research should focus on overcoming these challenges by advancing adaptive learning systems, optimizing real-time data processing, and ensuring ethical and responsible use of powered biosensors.

By addressing these issues, machine learning-assisted biosensors can fully unleash their potential, paving the way for more convenient, efficient, and personalized diagnostic tools. Smartphones equipped with machine learning algorithms will provide powerful solutions for direct interpretation of the testing results. For example, a mini-program can be created on a smartphone for data analysis to increase the usability and convenience, in which an algorithm is used to filter out abnormal frequency differences at the same concentration, fit the data into the model, and display the results on the screen. Additionally, given the increasing need for sensitive, stable, and multifunctional sensing devices, biosensors with machine learning abilities have good advantages in meeting the widespread needs of the medical industry. Thus, improving the performance of next-generation medical techniques requires the integration of machine learning with portable biosensors and other health-monitoring devices. This advancement will significantly promote the application of point-of-care testing biosensors in self-detection or home applications. Particularly, when artificial intelligence is combined with portable miniature optical biosensors, there is great potential for a point-of-care testing revolution in medical diagnosis. The advancement of nanotechnology, microfabrication, and flexible electronics technology will promote the development of compact wearable devices for continuous and real-time monitoring of biomarkers, enabling early detection of diseases in the personal medical field.

Generalizability is another challenge in the clinical application of machine learning models for AD diagnosis. In the reported models, the training datasets are well-representative samples of the clinical populations. They may include high similarities in the aspects of ethnicity distribution, genetic variation, age range, and environmental/lifestyle factors between the datasets and future patient populations. However, in real-life screening scenarios, it is expected that the vast majority of patients or donors will be non-AD people, resulting in a very imbalanced distribution of categories in the datasets. This highlights the necessity of implementing strict model validation strategies to provide reliability measurements for each prediction based on individual characteristics and similarity in the model training, such as *k*-fold cross-validation, leave-one-out validation, and testing on an external dataset.

In addition, the heterogeneity of AD will affect the accuracy of the machine learning models and the effectiveness of target detection. In training or testing datasets with potentially different pathological and/or clinical disease processes, there may be some rare disease variants present in the patient populations. The heterogeneity will also complicate the statistical differentiation of given biomarkers for potential AD variants, especially in patient populations with low mutation frequencies. In addition to providing predictions for AD, machine learning algorithms can also facilitate knowledge discovery by identifying the associations and patterns in data that would not be recognized without learning. The association and pattern may guide us to propose new pathological mechanisms or biomarkers. Nevertheless, the reliability of this inference is greatly dependent on the effectiveness of the computer models. Therefore, it requires a comprehensive critical analysis of the findings and strategies, such as multiple data sources and various machine learning techniques.

## Data Availability

No new data were created or analyzed in this study.
